# Children With Asthma Have Impaired Innate Immunity and Increased Numbers of Type 2 Innate Lymphoid Cells Compared With Healthy Controls

**DOI:** 10.3389/fimmu.2021.664668

**Published:** 2021-06-17

**Authors:** Banafshe Hosseini, Bronwyn S. Berthon, Malcolm R. Starkey, Adam Collison, Rebecca F. McLoughlin, Evan J. Williams, Kristy Nichol, Peter AB. Wark, Megan E. Jensen, Carla Rebeca Da Silva Sena, Katherine J. Baines, Joerg Mattes, Lisa G. Wood

**Affiliations:** ^1^ Priority Research Centre for Healthy Lungs, Hunter Medical Research Institute, University of Newcastle, Newcastle, NSW, Australia; ^2^ Priority Research Centre GrowUpWell, Hunter Medical Research Institute, University of Newcastle, Newcastle, NSW, Australia; ^3^ Department of Immunology and Pathology, Central Clinical School, Monash University, Melbourne, VIC, Australia; ^4^ Department of Respiratory and Sleep Medicine, John Hunter Hospital, Newcastle, NSW, Australia

**Keywords:** asthma, children, innate immune response, innate lymphoid cells, lung function

## Abstract

**Background:**

Asthma is the most frequent cause of hospitalisation among children; however, little is known regarding the effects of asthma on immune responses in children.

**Objective:**

The present study aimed to evaluate cytokine responses of peripheral blood mononuclear cells (PBMCs), PBMC composition and lung function in children with and without asthma.

**Methods:**

Using a case-control design, we compared 48 children with asthma aged 3-11 years with 14 age-matched healthy controls. PBMC composition and cytokine production including interferon (IFN)-γ, interleukin (IL)-1β, IL-5 and lL-6 following stimulation with rhinovirus-1B (RV1B), house dust mite (HDM) and lipopolysaccharide (LPS) were measured. Lung function was assessed using impulse oscillometry and nitrogen multiple breath washout.

**Results:**

The frequency of group 2 innate lymphoid cells were significantly higher in asthmatics and PBMCs from asthmatics had deficient IFN-γ production in response to both RV1B and LPS compared with controls (P<0.01). RV1B-induced IL-1β response and HDM-stimulated IL-5 production was higher in asthmatics than controls (P<0.05). In contrast, IL-1β and IL-6 were significantly reduced in response to HDM and LPS in asthmatics compared to controls (P<0.05). Children with asthma also had reduced pulmonary function, indicated by lower respiratory reactance as well as higher area of-reactance and lung clearance index values compared with controls (P<0.05).

**Conclusion:**

Our study indicates that children with asthma have a reduced lung function in concert with impaired immune responses and altered immune cell subsets. Improving our understanding of immune responses to viral and bacterial infection in childhood asthma can help to tailor management of the disease.

## Introduction

Asthma is the most prevalent chronic childhood condition (1, 2). Children with asthma frequently experience exacerbations, and the majority of exacerbations in children are associated with viral infections (3). While rhinoviruses (RVs) are the most frequent precipitants of virus-associated exacerbations in children (4), asthma exacerbations may also be triggered by invasive bacterial infection (5). Indeed, there is evidence showing strong associations between levels of household lipopolysaccharide (LPS) and asthma exacerbations (6–8) and reduced lung function (7). However, in children with asthma, a greater understanding of the mechanisms underlying the immune response to different stimuli, and factors contributing to increased risk of infection are required.

There are numerous immune cells, including neutrophils, eosinophils, natural killer (NK) cells, dendritic cells (DCs), lymphocytes as well as structural cells such as epithelial cells, that may contribute to an altered immune response in childhood asthma. Recent studies have demonstrated that innate lymphoid cells (ILCs) also have a key role in the development of virus-induced asthma exacerbations (9). Group 2 ILC (ILC2) secrete interleukin (IL)-5, IL-9 and IL-13 in response to IL-25 and IL-33 stimulation (9, 10), and circulating ILC2 levels are increased in adults with asthma. However, the role of ILC2 in childhood asthma is less clear.

The aims of this study were to compare cytokine responses of peripheral blood mononuclear cells (PBMCs) (including interferon (IFN)-γ, IFN-λ, IL-1β, IL-5 and IL-6) stimulated with RV1B, LPS and house dust mite (HDM) in asthmatic children with healthy controls and to characterise immune cell subsets (ILCs, eosinophils, neutrophils, lymphocytes, NK cells and DCs) in whole blood. We further investigated the relationship between immune cell subset populations and PBMC cytokine responses. Additionally, we aimed to compare the lung function parameters between children with and without asthma.

## Materials and Methods

### Study Design and Participants

This was an observational, case-control study including children with asthma aged 3-11 years (n=48) and healthy, age-matched controls with no previous diagnosis of asthma or history of respiratory conditions (n=14). Children with asthma were recruited *via* attendance to the emergency department or admission to the John Hunter Children’s Hospital and Maitland Hospital, following an exacerbation of asthma. This study includes baseline data obtained from asthmatic children participating in a 26-week clinical trial evaluating the effects of a high fruit and vegetable diet on asthma exacerbation (ACTRN12615000851561). Healthy controls were recruited *via* flyers placed in community centres and at the University of Newcastle (UoN). The study was conducted at the Hunter Medical Research Institute (HMRI), Newcastle, Australia, between September 2015 and March 2019. All participants were screened for eligibility prior to enrolment.

Inclusion criteria for children with asthma were physician diagnosis of asthma; recent exacerbation/s (≥1 exacerbation in past 6 months or ≥2 in the past 12 months) and stable asthma at the visit. Exclusion criteria included other respiratory conditions, diagnosed intestinal disorders, or consumption of nutritional supplements (in previous 4 weeks). Inclusion and exclusion criteria were the same for the control group, with the exception that controls had no history of asthma or wheeze. All subjects were consuming a low fruit and vegetable diet (≤3 serves of fruit and vegetables per day (assessed over past week). The study was approved by the HNEH Ethics Committee (15/06/17/4.03) and registered with the UON Human Research Ethics Committee. Written informed parental consent and child assent (where applicable), was obtained prior to participation in the study.

### Clinical Assessment

All participants fulfilling the inclusion criteria attended HMRI for clinical assessment and blood collection following a 12 hour overnight fast. Clinical assessments included anthropometry, nitrogen multi-breath washout (MBW), impulse oscillometry (IOS) and blood collection. For details, refer to supplement.

### Laboratory Methods

Immunoglobulin E (IgE) specific antibodies against 4 allergens (dust mites, mixed moulds, mixed grasses, and mixed animal epithelial) were measured in plasma using an ImmunoCAP Fluorenzyme assay (Pathology North, Newcastle, NSW Australia).

PBMCs were isolated from whole blood by density gradient method (11) using Ficoll-PaqueTM PLUS (GE Healthcare, Sydney, Australia) and cultured with and without RV1B, LPS or HDM for 48 hours. The concentrations of IFN-γ, IL-1β and IL-6 in the culture supernatants were analysed using bead-based multiplex assay (BD Bioscience, Sydney, Australia) and IFN-λ and IL-5 concentrations in the culture supernatants were measured using high-sensitivity commercial ELISA assays (R&D Systems, Sydney, Australia) as per the manufacturer’s recommendations.

Quantification of major immune cell subsets in whole blood, including eosinophils and neutrophils, T lymphocytes, DCs, NK cells plus B cells and ILCs was performed by multi-parametric flow cytometry using the Fortessa LSR-X20 (BD Biosciences, Sydney, Australia).

For full laboratory analysis methods see supplement.

### Statistical Analysis

Statistical analyses were performed using STATA 15 (StataCorp, College Station, Texas, USA). Data are reported as mean ± standard deviation or median (interquartile range). Significant differences between groups were determined using independent *t*-test (parametric data) or Wilcoxon Rank Sum tests (non-parametric data). Subgroup analyses on immune responses of PBMCs were performed on a subset of participants with negative RAST results (no history of allergy) as well as those with no history of OCS use. In adjusted comparisons, age, weight and height were adjusted using General Linear Model. Pearson’s Chi-squared test or Fisher’s exact test (expected cell sizes< 5) were used to test equality of proportions between groups. Exploratory analysis between immune cell subset populations and PBMC cytokine responses were assessed using Spearman’s correlations. Significance was accepted when *P*<0.05.

## Results

### Subject Characteristics

Demographic and clinical characteristics of the children with asthma and healthy controls enrolled in the study are listed in **Table 1A**. There were no significant differences in age, weight, height, BMI and BMI z score among the asthmatic children and healthy controls. The proportion of males was significantly higher in children with asthma compared to the healthy controls. Furthermore, the history of eczema (60.4% versus 0%, *P<0.0001)* or hayfever (60.4% versus 14.3%, *P<0.01)* was greater in the asthma group compared with the control group. Similarly, there were more children with history of food allergy in the asthmatics than in healthy controls, which appeared to be marginally significant (P=0.056). There were also more subjects with asthma that had a positive RAST result to dust mite (allergen specific IgE 15-50 kUA/L) (*P*<0.001).

**Table 1a:** Subject demographics and clinical characteristics.

Variable	Asthma (n=48)	Healthy controls (n=14)		*P-*value
**Gender (Male: Female)**	35:13		5:9	**0.023**
**Age (years)**	5.25 (3.82, 6.99)		6.58 (4.99, 8.38)	0.136
**Age 3-6 years, n (%)**	32 (66.7)		9 (64.3)	1.00
**Age 7-11 years, n (%)**	16 (33.4)		5 (35.7)	
**Weight (kg)**	21.50 (16.70, 25.75)		23.55 (18.70, 30.70)	0.215
**Height (cm)**	116.25 (103.45, 124.70)		122.16 (115.50, 133.30)	0.079
**BMI z-score**	0.05 ± 0.19		-0.04 ± 0.05	0.814
**BMI percentile**	51.70 ± 31.63		50.14 ± 33.36	0.872
**Current food allergy, n (%)**	11 (22.9)		0 (0)	0.056
**History of Eczema** ^*^, **n (%)**	29 (60.4)		0 (0)	**<0.001**
**History of Hayfever** ^^^, **n (%)**	29 (60.4)		2 (14.3)	**0.002**
**Positive RAST Results^‡^, n (%)**	n=47		n=13	
Dust Mite	29 (61.7)		0 (0)	**<0.001**
Animal mix	4 (8.5)		0 (0)	0.413
Grass mix	5 (10.6)		0 (0)	0.335
Mould mix	1 (2.1)		0 (0)	0.810

Data are presented as mean ± SD or median (interquartile). BMI z-scores and percentiles were calculated using the Centre for Disease Control and Prevention (CDC) 2000 Growth Charts. Difference between groups analysed by the Wilcoxon Rank sum test (non-parametric data), two-sample t-test (parametric data) or Pearson’s Chi-squared test/Fisher’s exact test (testing equality of proportions). P<0.05 considered statistically significant. *Based on parental response to “Has your child ever had eczema?” ^Based on parental response to “Has your child ever had a problem with sneezing, or a runny or blocked nose when he/she DID NOT have a cold or the flu?”. ^‡^Plasma allergen-specific immunoglobulin E level between 15.0-50 kUA/L. Bold values indicate statistically significant difference (P<0.05) noted between the two groups.

Children with asthma were well-controlled at the time of sample collection [median asthma control score of 24 (21, 25)] (**Table 1B**). Of 48 subjects in the asthmatic group, 58.3% had ≥1 hospital admission for acute asthma exacerbation in the previous 12 months. Asthma medication use in the previous 12 months is presented in **Table 1B**.

**Table 1b:** Clinical characteristics of children with asthma.

Characteristics	Asthma (n=48)
Asthma control score, median (IQR)	24 (21, 25)
≥1 hospital admission due to exacerbation in previous 12 months, n (%)	28 (58.3)
**Medication use, n (%)**	
OCS intermittent	21 (43.7)
ICS or ICS/LABA combination	33 (68.7)
ICS intermittent*	7 (14.5)
ICS maintenance^^^	22 (45.8)
ICS/LABA maintenance ^^^	4 (8.3)
SABA only	12 (25.0)

IQR, interquartile; OCS, oral corticosteroids; ICS, inhaled corticosteroids; LABA, long-acting β2-agonist; SABA; short-acting β2-agonist. *Reported to have been taken intermittently or on an as-needed basis. ^Reported to have been taken for most of the previous 12 months.

### *Ex Vivo* Stimulation of Cytokine Production by PBMCs

#### Cytokine responses of PBMCs to Human Rhinovirus-1B stimulation

**Figure 1:**
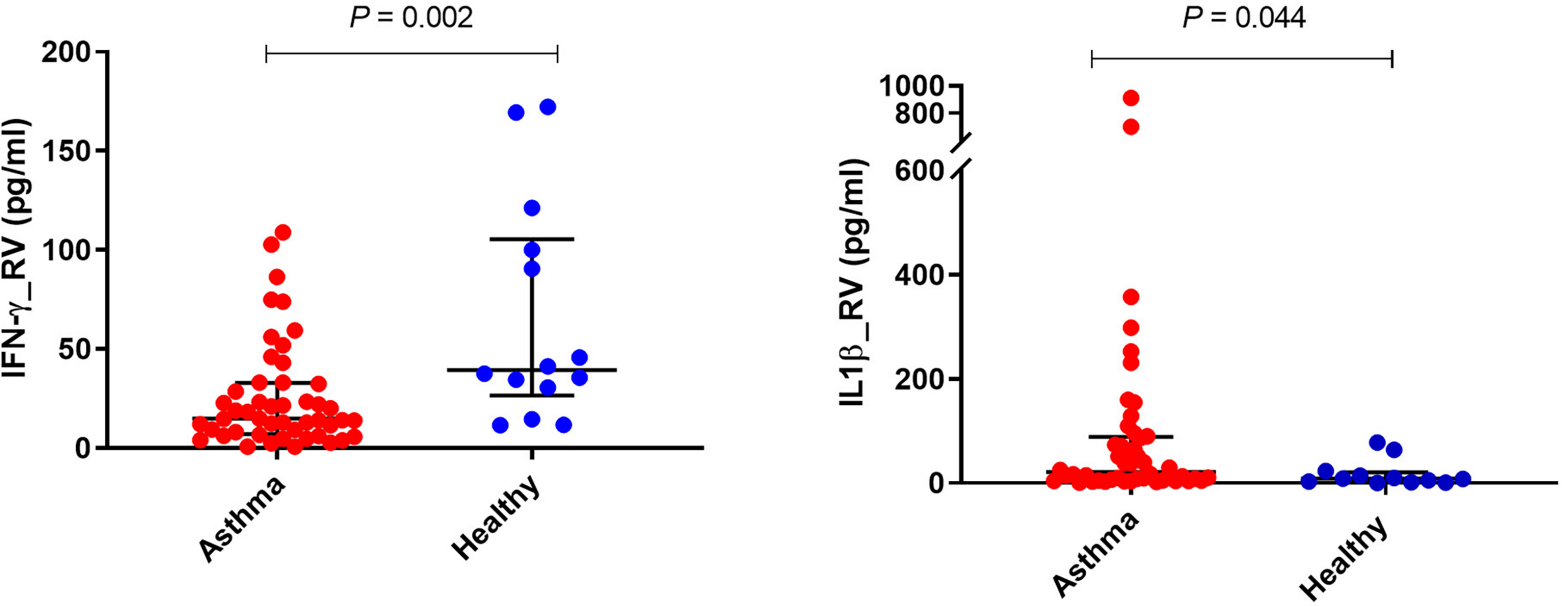
Effects of RV1B stimulation on cytokine responses of PBMCs. PBMCs from children with asthma (n=48) and healthy controls (n=14) were exposed to media or RV1B (MOI=20) for 48h. Bars represent median (interquartile range). Data adjusted for the levels in uninfected PBMCs (control). Plot represents median with interquartile ranges and individual’s values are represented by dots. PBMC, peripheral blood mononuclear cells; RV, rhinovirus; IFN, interferon.

PBMCs from children with asthma stimulated with RV1B produced significantly less IFN-γ compared with PBMCs from healthy controls (*P*<0.01) (**Figure 1** and **Table 2**). A similar trend was observed for IFN-λ, although the difference did not reach statistical significance. In contrast, the concentrations of IL-1β in the supernatants of PBMCs infected with RV1B were significantly higher in children with asthma than in the healthy controls (*P*<0.05) (**Figure 1** and **Table 2**). The RV1B induced IL-6 response was also higher in asthmatics than healthy controls; however, this was not significant.

**Table 2:** Cell supernatant cytokine secretion of PBMCs in response to different stimuli in children with asthma and age-matched non-asthmatic group.

Cytokine *(pg/ml)*	Asthma (n=48)	Healthy controls (n=14)	*P*-value
*PBMCs stimulated with Rhinovirus-1*			
**IFN-γ**	14.88 (7.38, 32.72)	39.40 (30.59, 100.1)	**0.002**
**IFN-λ**	5.13 (1.87, 13.41)	6.63 (3.22, 89.46)	0.192
**IL-1β**	21.25 (5.08, 88.15)	8.29 (2.25, 18.54)	**0.044**
**IL-5**	0.51 ± 0.27	0.48 ± 0.26	0.649
**IL-6** (*ng/mL)*	1.43 (0.31, 8.93)	0.48 (0.19, 2.46)	0.372
*PBMCs stimulated with House Dust Mite*			
**IFN-γ**	1.11 (0.77, 1.35)	0.99 (0.78, 1.14)	0.932
**IFN-λ**	2.18 (1.05, 4.55)	3.84 (2.1, 6.08)	0.159
**IL-1β**	14.25 (8.88, 49.04)	76.99 (52.88, 114.88)	**<0.001**
**IL-5**	6.62 (1.10, 22.86)	0.93 (0.54, 1.75)	**0.005**
**IL-6** (*ng/mL)*	2.43 (1.00, 12.16)	30.90 (19.58, 56.81)	**<0.001**
*PBMCs stimulated with Lipopolysaccharide*			
**IFN-γ**	1.66 (1.06, 5.53)	41.14 (16.29, 55.50)	**<0.001**
**IFN-λ**	2.58 (0.88, 7.12)	2.21 (1.33, 3.06)	0.655
**IL-1β**	1888.58 (1173.98, 5863.56)	10681.23 (7967.57, 15169.19)	**<0.001**
**IL-5**	0.53 (0.25, 0.78)	0.60 (0.37, 0.79)	0.711
**IL-6** (*ng/mL)*	73.26 (47.64, 119.08)	127.31 (82.20, 146.08)	**0.021**

Data are presented as median (interquartile range) or mean ± SD. All variables adjusted for the levels in uninfected PBMCs (control). Difference between groups analysed by Wilcoxon Rank Sum test (non-parametric data) or two-sample t-test (parametric data). PBMC, peripheral blood mononuclear cells; IFN, interferon; IL, interleukin. Bold values indicate statistically significant difference (P<0.05) noted between the two groups.

#### Cytokine responses of PBMCs to House Dust Mite stimulation

**Figure 2:**
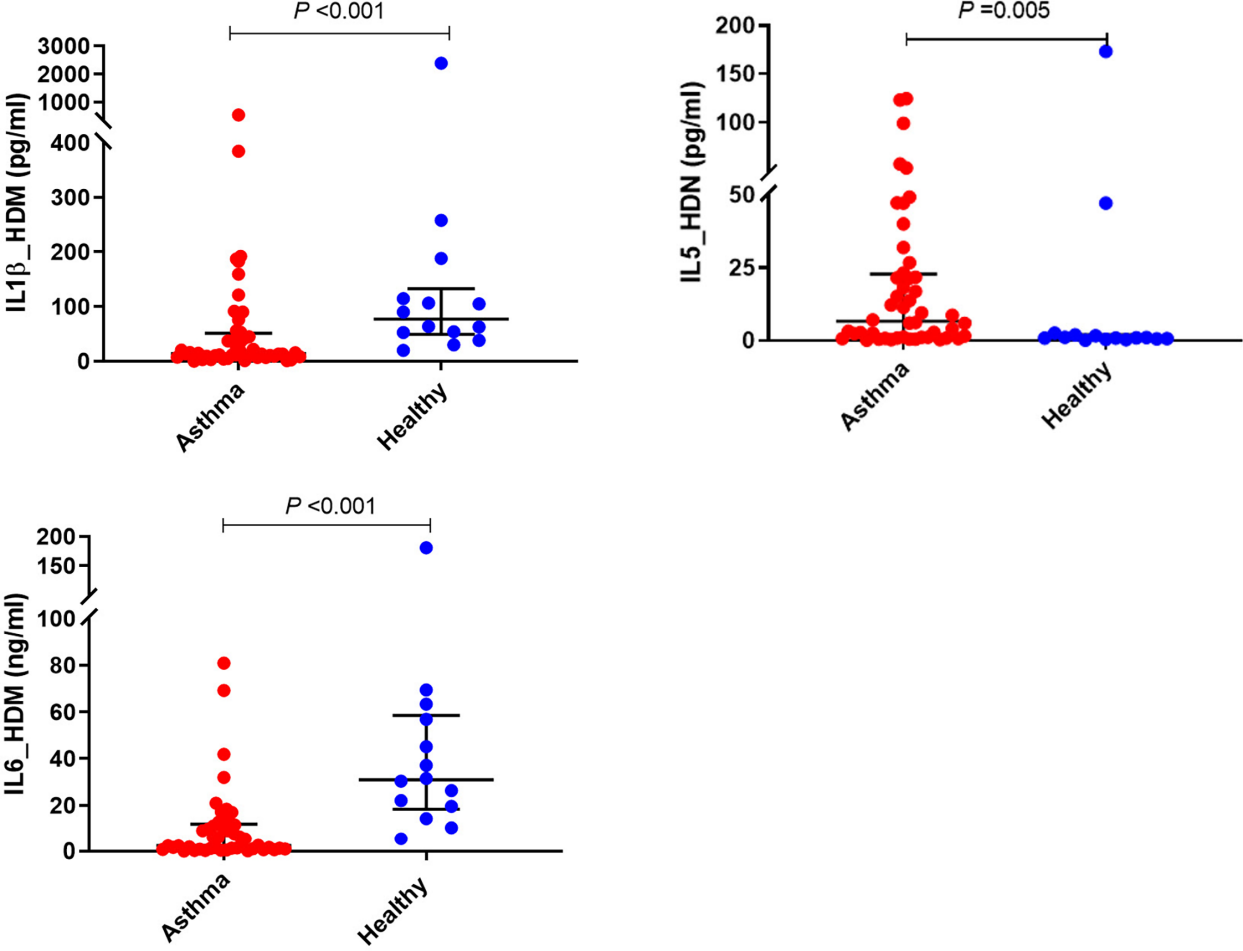
Effects of HDM stimulation on cytokine responses of PBMCs. PBMCs from children with asthma (n=48) and healthy controls (n=14) were exposed to media or HDM for 48h. Bars represent median (interquartile range). Data adjusted for the levels in uninfected PBMCs (control). Plot represents median with interquartile ranges and individual’s values are represented by dots. PBMC, peripheral blood mononuclear cells; HDM, house dust mite; IL, interleukin.

HDM-induced IL-1β and IL-6 production were significantly lower in children with asthma than healthy controls (*P*<0.001) (**Figure 2** and **Table 2**). In contrast, the production of type 2 cytokine, IL-5, following HDM exposure was significantly higher in asthmatic children than in age-matched healthy controls (*P*<0.01) (**Figure 2** and **Table 2**). However, there were no significant differences in HDM-induced IFN-γ, and IFN-λ production in PBMCs obtained from asthmatic children compared with those from healthy controls (*P>0.05)*.

#### Cytokine responses of PBMCs to Lipopolysaccharide stimulation

**Figure 3:**
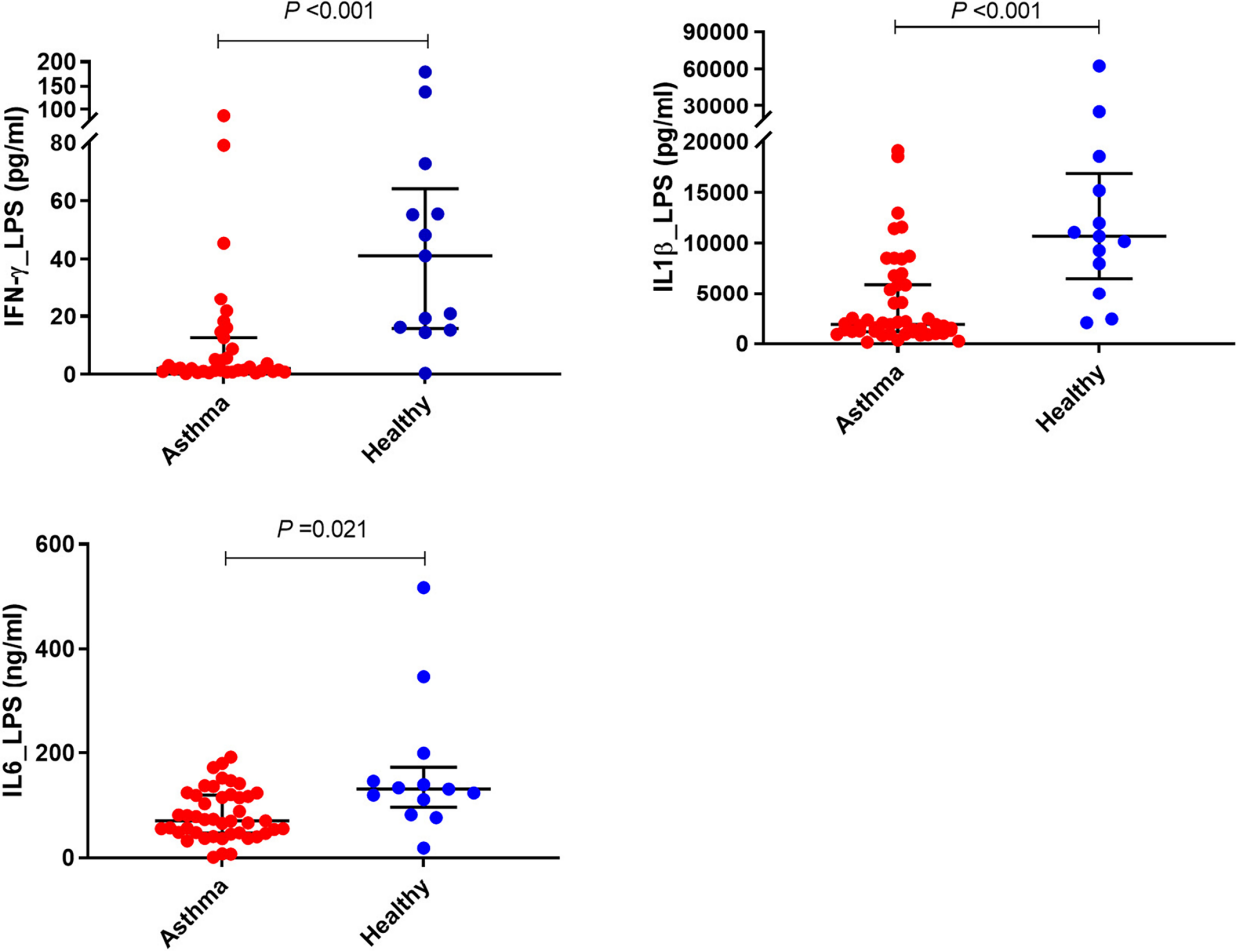
Effects of LPS stimulation on cytokine responses of PBMCs. PBMCs from children with asthma (n=48) and healthy controls (n=14) were exposed to media or LPS for 48h. Bars represent median (interquartile range). Data adjusted for the levels in uninfected PBMCs (control). Plot represents median with interquartile ranges and individual’s values are represented by dots. PBMC, peripheral blood mononuclear cells; LPS, lipopolysaccharide; IFN, interferon.

There was significantly less IFN-γ produced by LPS stimulated PBMCs from children with asthma compared with those from the controls (*P*<0.001) (**Figure 3** and **Table 2**); however, no significant difference was found in LPS- induced IFN-λ production in PBMCs obtained from children with asthma compared with PBMCs from controls. Furthermore, PBMCs stimulated with LPS produced the same pattern of IL-1β and IL-6 response observed for HDM with PBMCs from asthmatic children producing significantly lower IL-1β and IL-6 compared with PBMCs from healthy controls (*P*<0.001 and *P*<0.05, respectively) (**Figure 3** and **Table 2**).

#### Subgroup analyses

A subgroup analysis on children with negative RAST results was performed on 19 children with asthma and 13 healthy controls. Production of IFN-γ in response to both RV1B and LPS was lower in asthmatic children than healthy controls (*P*= 0.018 and *P*= 0.009, respectively). Additionally, HDM stimulated IL-1β and IL-6 production (*P*=0.002 and *P*= 0.001, respectively) and as well as LPS induced IL-1β release were found to be lower in asthmatics compared to the healthy controls (*P*=0.005) (**Table S3**).

A subgroup analysis was also carried out on a subset of participants with no history of OCS use (asthmatics=27, healthy controls= 14). Children with asthma had deficient IFN-γ production in response to both RV1B and LPS compared with healthy controls (P=0.003 and P<0.0001, respectively). Moreover, IL-1β and IL-6 production in response to both HDM (*P <*0.001 and P<0.001, respectively) and LPS (*P <*0.001, *P*=0.029, respectively) were found to be lower in asthmatic children than in their age-matched counterparts (**Table S4**).

### Peripheral Whole Blood Immune Cell Profiles

**Figure 4:**
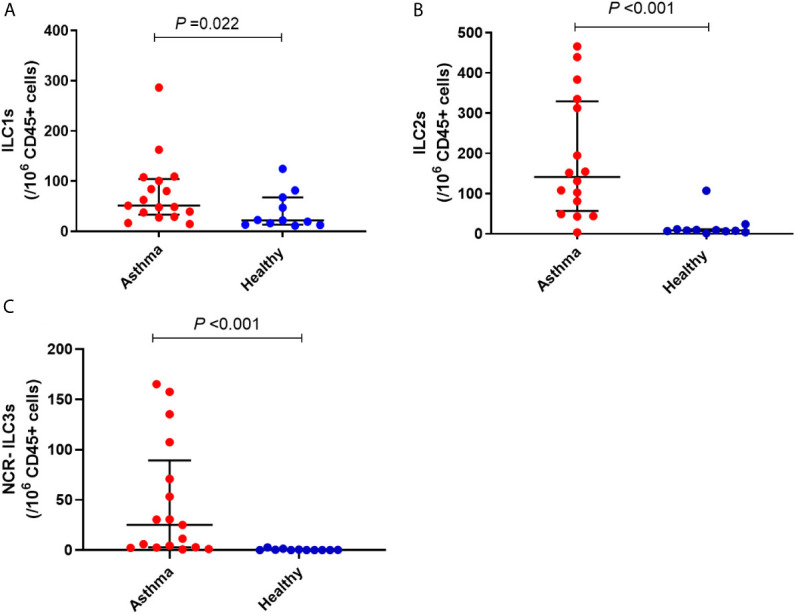
Frequency of **(A)** ILC1, **(B)** ILC2 and **(C)** NCR- ILC3 in children with asthma and healthy controls. Bars represent median (interquartile range). Immune cell phenotyping was performed in whole blood using a lyse-wash procedure. Cells are per 10^6^ CD45^+^ cells. Plot represents median with interquartile ranges and individual’s values are represented by dots. ILCs, innate lymphoid cells; NCR, natural cytotoxicity receptor.

Immune cell subset frequency was assessed in PBMCs from 17 asthmatics and 11 healthy children. Asthmatic children showed a significantly higher number of ILC1 and ILC2 compared to healthy controls [(51.09 (39.04, 100.62) *versus* 21.91 (13.04, 67.60), *P*<0.05 and 151.80 (81.16, 335.17) versus 8.40 (6.52, 11.46), *P*<0.001, respectively]. Moreover, while there were no significant differences in numbers of ILC3 with natural cytotoxicity receptor (NCR+) between the two groups, the number of circulating ILC3 NCR- was significantly higher in children with asthma compared to healthy controls [25.00 (2.52, 70.99) *versus* 0.0 (0, 0.53), *P*<0.001] (**Figure 4** and **Table 3**). The frequency of granulocytes, DCs, NK cells and lymphocytes were similar in the two groups (**Table 3**).

**Table 3:** Frequency of circulating immune cell subsets in children with asthma and age-matched non-asthmatic group.

Cell Subsets	Asthma (n=17)	Healthy controls (n=11)	*P*-value*
**Innate lymphoid cells^1^**			
ILC1	51.09 (39.04, 100.62)	21.91 (13.04, 67.60)	**0.022**
ILC2	151.8 (81.16, 335.17)	8.40 (6.52, 11.46)	**<0.001**
ILC3 NCR-	25 (2.52, 70.99)	0 (0, 0.53)	**<0.001**
ILC3 NCR+	0 (0, 0.69)	0 (0, 0)	0.052
**Granulocytes**			
Eosinophils	111914.8 ± 142033.3	63698.76 ± 34578.89	0.141
Neutrophils	280270.5 ± 137243	345993 ± 169330.6	0.865
**Dendritic cells**			
BDCA-1	30476.19 (16366.61, 37001.29)	6503.078 (1912.066, 43572.04)	0.111
BDCA-3	4444.444 (2646.28, 21046.3)	14217.13 (1572.327, 73949.58)	0.706
pDCs	26904.76 (19498.61, 57909.61)	16758.32 (3783.01, 40094.34)	0.352
**NK cells**	41905.82 (33153.01, 68353.91)	38138.79 (13537.55,56780.51)	0.543
**B cells**	432004.3 (412312.2, 634458.6)	287007.8 (234278.1, 486687)	0.068
**T cells**			
CD4^+^ T cells	528823.5 (517112.3, 591268.7)	562661.8 (502188.2, 591235.2)	0.724
Activated CD4^+^	34910.71 (27368.62, 46767.48)	34416.15 (25020.48, 79951.78)	0.795
Treg cells	36234.1 (34276.74, 39402.04)	32471.75 (27356.96, 40535.74)	0.249
CD8^+^ T cells	189591.7 ± 140576.2	206728.8 ± 104836.4	0.732
Activated CD8^+^	674.48 (247.83, 1730.77)	1056.32 (684.84, 4197.76)	0.094
TCR-beta T cells	742566.8 (683641.6, 832381)	768274.4 (729063.5, 838161.9)	0.759
γδ-T cells	81940.52 (58436.9, 115443.6)	60867.0 (48278.07, 89825.17)	0.249

Data are presented as mean ± SD or median (interquartile range). ^1^Innate lymphoid cells and granulocytes are per 10^6^ CD45^+^ cells. Dendritic cells are per 10^6^ human leukocyte antigen^+^ cells. B cells, T cells and NK cells adjusted are per 10^6^ CD3^+^ cells. *Difference between groups analysed by Wilcoxon Rank Sum test (non-parametric data) or two-sample t-test (parametric data). P < 0.05 considered statistically significant. ILCs, innate lymphoid cells; NCR, natural cytotoxicity receptor; BDC, blood dendritic cells; PDC plasmacytoid dendritic cells; NK cells, natural killer cells; TCR-β, T cell receptor-β; Treg cells, T regulatory cells. Bold values indicate statistically significant difference (P<0.05) noted between the two groups.

### Associations

Correlation analysis between peripheral blood immune cell numbers and cytokine responses of PBMCs revealed an inverse association between the frequency of ILC1 and LPS-induced IL-1β (r_s_=-0.45, *P*=0.030). Furthermore, the frequency of ILC2 was inversely correlated with LPS-induced IL-1β and IL-6 (r_s_=-0.43, *P*=0.035; and r_s_=-0.45, *P*=0.030, respectively), and HDM-induced IL-6 (r_s_=-0.54, *P*=0.006). Inverse associations were also found between the prevalence of ILC3 NCR- and RV1B-induced IFN-γ (r_s_=-0.48, *P*=0.019), HDM-induced IL-6 (r_s_=-0.77, *P*<0.0001), and LPS-induced IL-1β (r_s_=-0.48, *P*=0.018). Moreover, the frequency of B cells was inversely associated with RV1B-induced IFN-λ (r_s_=-0.45, *P*=0.035). In addition, the frequency of TCR- β cells was positively associated with LPS-induced IL-1β (r_s_=0.44, *P*=0.031).

### Lung Function Assessments

#### Impulse Oscillometry

IOS variables included in the analysis were airway reactance (Xrs), respiratory resistance (Rrs) (all measured at 5, 10, 15 and 20 Hz), as well as respiratory impedance measured at 5 Hz (Z5), and reactance area (Ax). Children with asthma demonstrated lower reactance at 5, 10, 15 and 20 Hz compared with healthy controls (**Table 4**). Whereas, Ax values were greater in asthmatics than in healthy controls (**Table 4**). No difference was found in respiratory resistance and respiratory impedance between the two groups. After adjusting for age, weight and height, the difference between airway resistance at 5Hz and 20Hz (R_5_-R_20_) was significantly higher in asthmatics than in healthy controls (*P*<0.05). Additionally, the differences in airway reactance remained significant after adjusting for age, weight and height, while Ax value was no longer different between the groups.

**Table 4:** Comparison of lung function parameters between children with asthma and age matched non-asthmatic group.

Variable	Asthma (n=27)	Healthy controls (n=13)	*P-*value	Adjusted P*
*X*_5_ Hz z-score	0.56 ± 0.45	0.93 ± 0.53	**0.028**	**0.043**
*X_10_* Hz z-score	0.63 (-0.18, 0.83)	0.96 (0.64, 1.61)	**0.031**	**0.034**
*X_15_* Hz z-score	1.47 (0.65, 2.23)	2.28 (1.87, 2.72)	**0.009**	**0.012**
*X_20_* Hz z-score	-0.23 ± 1.24	0.64 ± 1.06	**0.036**	**0.023**
*R*_5_ Hz z-score	-0.37 ± 0.91	-0.52 ± 0.83	0.624	0.622
*R_10_* Hz z-score	-0.22 ± 0.99	-0.30 ± 0.73	0.810	0.713
*R_15_* Hz z-score	-0.39 (-0.89, 0.30)	-0.35 (-0.63, 0.49)	0.711	0.986
*R_20_* Hz z-score	-0.04 ± 1.06	0.12 ± 0.61	0.601	0.664
*R_5_-R_20_*	-0.33 ± 0.51	-0.65 ± 0.44	0.064	**0.036**
*Z*_5_ Hz z-score	-0.42 ± 0.87	-0.60 ± 0.80	0.533	0.535
Ax (kPas/L)	1.01 (0.57, 1.67)	0.57 (0.20, 0.73)	**0.013**	0.085
LCI_2.5_	7.65 ± 1.36	6.56 ± 0.56	**0.034**	**0.006**
FRC	1.27 (0.88, 1.54)	1.16 (1.07, 1.34)	0.953	0.385

Lung function was measured using Impulse Oscillometry and Nitrogen Multiple- Breath washout. Data are presented as mean ± SD or median (IQR). Difference between groups analysed by Wilcoxon Rank Sum test (non-parametric data) or two-sample t-test (parametric data). Patients with missing and/or invalid data were excluded for each variable. *Age, weight and height were adjusted using General Linear Model. P<0.05 considered statistically significant. X, respiratory reactance; R, respiratory resistance; Z, respiratory impedance; AX, area of reactance; LCI, lung clearance index; FRC, Functional residual capacity. Bold values indicate statistically significant difference (P<0.05) noted between the two groups.

#### Nitrogen Multiple-Breath Washout

Lung clearance index (LCI)_2.5_ values were on average 1.09 units (95% confidence interval: 0.09- 2.09, *P*< 0.05) higher in children with asthma compared with healthy controls (**Table 4**). No significant difference was detected in functional residual capacity between the groups. Adjusting for age, weight and height did not alter the results.

## Discussion

For the first time, this study has demonstrated that children with asthma had deficient IFN-γ production in response to both RV1B and LPS infection, compared with the healthy controls. RV1B induced IL-1β response was higher in asthmatics than healthy controls. HDM-stimulated IL-5 production was also significantly greater in asthmatic children than in healthy controls. In contrast, both IL-1β and IL-6 production were significantly lower in response to HDM and LPS in children with asthma. Furthermore, the frequency of ILC1, ILC2, and ILC3 NCR- were significantly higher in children with asthma compared to healthy controls, while other immune cells such as granulocytes, DCs, B cells and T-cells were present in whole blood in similar numbers. These results indicate that innate immune dysfunction in asthma is not limited to adults and may explain the increased susceptibility of asthmatic children to viral and bacterial respiratory infections.

In the present study, we observed that *ex vivo* RV1B infection of PBMCs from asthmatic children resulted in >2.6 times less IFN-γ production compared with PBMCs from healthy controls. Whereas, RV1B-induced IL-1β production was 2.5-fold higher in asthmatic cells than in healthy controls, which suggests inflammasome induction (12). Our results suggest that diminished IFN-γ production, as well as overproduction of inflammatory cytokines (IL-1β) in children with asthma, may be involved in their high susceptibility to lower airway symptoms caused by RV infection. These findings are in concordance with another study in pre-school children (13) that showed reduced serum IFN-α levels in children with asthma compared to healthy controls. In line with our findings, numerous studies in adults reported that following RV exposure PBMCs from adult patients with asthma produced lower levels of IFN-γ compared with cells from healthy controls (14, 15).

IFNs can decrease susceptibility of host cells to viral infection, and an inverse association between IFN-γ production and viral load has been shown previously (3, 16). The antiviral activities of IFNs are mediated directly through the inhibition of viral replication in cells and indirectly through the induction of cytokines and chemokines, which results in recruitment of NK cells as well as CD4 and CD8 T cells (17). Diminished antiviral IFN responses in asthma could be the main mechanism for enhanced susceptibility to respiratory viral infection (18). This mechanism could be an explanation for asthmatic patients having more severe lower respiratory tract symptoms and declines in lung function of greater duration and severity following RV infection (18).

IFN-γ also appears to be a key mediator of LPS-induced immune responses (19). We observed a deficient IFN-γ response to LPS in asthmatic children compared with age-matched healthy controls. Similarly, Contoli *et al.* showed primary bronchial epithelial cells and alveolar macrophages from adult asthmatic patients produced lower levels of IFN-λ following LPS stimulation than healthy controls (5). IL-6 and IL-1β production by PBMCs in response to HDM and LPS were impaired in children with asthma in our study. There is apparent controversy about the immune responses to LPS in patients with asthma. A previous study showed that following LPS exposure, PBMCs from adult asthmatic patients produced more IL-1β than cells from healthy controls (20), while, another study in adults reported that asthmatic patients have defective innate immune responses to LPS demonstrated by lower LPS-induced IL-1B response in asthmatics compared to healthy participants (21). Recent studies have highlighted the role of toll-like receptors (TLRs) in the innate and adaptive immune responses. TLRs are involved in the initial immune response to pathogens or environmental stimuli and are broadly expressed by a variety of tissues and cell types (22, 23). Previous studies have demonstrated an association between TLRs and the pathogenesis of asthma (22). One of the well-characterised TLRs that can recognise ligands such as LPS and HDM is TLR4. It has been shown that the expression of TLR4 on PBMCs is diminished in asthmatic patients (22). Our finding of a significantly lower level of IL-6 and IL-1B in response to HDM and LPS in the asthmatic group is in concordance with the previous study that reported lower expression of TLR4 in asthma (22).

This study also demonstrated that HDM-stimulated IL-5 release was significantly higher in children with asthma than in healthy controls. This is in consistent with previous studies that reported children and adolescents with asthma had enhanced TH2 cytokines responses to HDM in comparison with healthy controls (24–26). Previous research suggested that HDM can trigger a TH2-type response in the T cells that specifically recognize the allergen but that allergen-responsive T cells might be lower in healthy subjects than in patients with asthma. It can be suggested that the impaired inflammatory response observed in patients with asthma is due to an imbalance between type I and type II cytokines.

There are several additional points that warrant consideration. The number of children with sensitization to dust mite was significantly higher in children with asthma compared with healthy controls (61.7% vs. 0%, respectively). Increased IgE antibodies to allergens was found to be correlated with increased risk for lower respiratory tract symptoms with viral infections such as rhinovirus in patients with asthma (16). To examine whether allergy status is one of the causes of the observed significant differences in immune responses of PBMCs, we performed a subgroup analysis on children with negative RAST results (n= 32) and differences in the following variables remained statistically significant between the two groups: RV1B induced IFN-γ (*P*= 0.018), HDM stimulated IL-1β and IL-6 production (*P*=0.002, *P*= 0.001, respectively) and as well as LPS induced IFN-γ and IL-1β release (*P*= 0.009 and *P*=0.005, respectively) (**Table S3**). These findings confirm that the deficient IFN responses observed in children with asthma is not related to their allergy status. However, it should also be noted that no significant difference was observed in HDM-specific IL-5 responses between the two groups after adjusting for allergy status. Our results indicate that HDM-induced IL-5 production can be considered as a predictor for the presence of atopy in children with asthma, as has been suggested by others (27). Additional questions of concern relate to the effects that corticosteroids may have on the observed differences in immune responses of PBMCs. Prior studies have reported immune-suppressive characteristics of steroids (28, 29). Twenty-one children in asthma group (43.7%) used intermittent oral corticosteroids, while none of the healthy controls used corticosteroids for any health conditions. Our subgroup analysis of subjects with no history of OCS use (n=41) revealed the following: IFN-γ production in response to both RV1B and LPS remained to be significantly lower in children with asthma compared with healthy controls (P=0.003 and P<0.0001). Additionally, IL-1β and IL-6 production in response to both HDM and LPS were remained lower in asthmatic children than in their age-matched counterparts (*P <*0.001) (**Table S4**). These findings are in line with previous *in vitro* studies that reported corticosteroids inhibit viral-induced cytokines but do not inhibit interferons (30). However, the interaction between corticosteroids and virus infection is controversial and some studies demonstrated that treatment with corticosteroids induced viral replication in the epithelial cells by suppressing type I and type III IFN production (31). It is also worth noting that in our study there was an apparent increase in RV1B induced IFN-λ production in subjects with no history of OCS; however, this was not significantly different between the two groups. These data indicate the need to further *in vivo* and *in vitro* studies in the role of corticosteroids in innate immune responses and activation of immune cells in asthma.

We also measured the frequency of whole blood immune cell subsets and found a higher prevalence of ILC1, ILC2, and ILC3 NCR- in children with asthma compared to healthy controls. These findings are consistent with previous studies that also reported higher prevalence of ILC2 in blood from adult asthmatic patients than healthy controls (9, 10, 32), which was associated with worse asthma control (10). However, in another study, ILC2 were on average 50% lower in the blood of children with acute asthma compared with healthy controls (33). Compelling evidence indicates that ILCs have significant roles in asthma development, specifically virus-induced asthma (9), as they link the innate and the adaptive immune responses within the hypersensitive airway (10). The airway epithelial cells, as the first natural barrier, protect the body from external antigens. In response to pathogen recognition, allergen exposure or viral infection, epithelial cell-derived cytokines such as IL-25 and IL-33 are released. Following secretion, IL-25 and IL-33 can bind to their receptors on the surface of ILC2 and affect the growth and proliferation of these cells (9, 34). Activated ILC2 cells are potent secretors of Th2 cytokines (e.g., IL-5, IL-13) (9, 34), which results in airway hyperactivity, mucus overproduction, airway smooth muscle constriction as well as airway remodelling (35).

In an exploratory analysis, we examined the relationship between peripheral blood immune cell numbers and cytokine responses of PBMCs to RV1B, HDM, and LPS stimulation. We found that the number of ILC2 and ILC3s NCR- was inversely correlated with HDM-induced IL-1β. LPS-induced IL-1β was found to be inversely correlated with ILC1s, ILC2 and ILC3s NCR-, while, positively associated with TCR+ cells. Inverse correlations were also found between ILC2 and IL-6 from LPS-stimulated PBMCs, as well as between ILC3s NCR- and IFN-γ production in response to RV1B stimulation. These findings suggest that increased circulating ILCs, in particular, ILC2 might be involved in dysregulated innate immune responses in asthmatic children. Additionally, we found that the frequency of B cells was inversely associated with RV1B-induced IFN-λ. This inverse correlation confirms the previous observation that virus-induced IFN-λ can reduce proliferation of B cells in a dose-dependent manner (36). To our knowledge, this is the first study to suggest a relationship between whole blood immunophenotype and innate immune responses of PBMCs. Previous research has explored the association between cord blood immune cell subsets and airway immune mediators and reported a positive association between activated CD4 and CD8 T cells and TNF-α, while regulatory T cells and CD4 T cells were reported to be inversely correlated with IL-1β (37).

Our study also revealed that compared with healthy controls, asthmatic children had lower lung reactance (X_rs_) values, while area of reactance was found to be significantly higher in asthmatics than in healthy controls. Moreover, after adjusting for age, weight and height, asthmatic children had a greater R_5_-R_20_ value compared with controls. This is consistent with previous studies that reported peripheral airway IOS indices (including A_x_ and R_5_-R_20_) were correlated with asthma control in both children and adults (38–41). Our findings are also in line with previous research showing that X_rs_ predicted values were significantly lower in asthmatics than in controls (42). In a cohort of 162 children aged 2-5 years, lower z-scores of reactance X_5_ were observed in persistent asthmatics compared with children with intermittent asthma (43). Our results showed that functional impairment of the airways is present, even in young children with stable asthma. The ability to predict loss of asthma control in children can decrease asthma related mobility and mortality. Thus, IOS can be used to identify paediatric patients with stable asthma who are at risk of losing asthma control.

The measurements from nitrogen MBW showed that, as expected, LCI_2.5_ was higher in asthmatic children than in healthy controls. These results are in agreement with previous research (44, 45). Baseline LCI was found to be significantly higher in school-aged children with asthma compared with healthy age-matched controls (40). Similarly, another study demonstrated that clinically stable paediatric patients with asthma had a significantly greater LCI value compared to healthy controls, which persisted after salbutamol use (41). These findings suggest that ventilation inhomogeneity is present in the airways of asthmatic children even in those with stable asthma. LCI has been found be useful in the recognition of early lung disease as well as in the prediction of lung function in children (46–49). Further studies are needed to assess the role of LCI in tracking the progression of early airway remodelling in children with asthma.

There are several limitations to our study, including the relatively small sample size of the control group. The rate of recruitment was lower than we had anticipated, with objections to the venous blood draw being the primary reason for parental refusal. Although, the sample size in this study was comparable to similar studies conducted in adults (50, 51) and children (52). Nonetheless, the study was adequately powered to detect important differences in circulating ILC subsets in children with asthma compared with healthy controls, and associations between immune cell subsets and responses to virus, HDM and LPS exposure, which are important observations.

A key strength of the present study is that the children with and without asthma were matched for age, which is known to affect immune responses (53). Additionally, asthma was defined by a doctors diagnosis (54). Another strength of this study is the exploration of the associations between whole blood immunophenotype and innate immune responses of PBMCs for the first time in children with asthma.

In summary, our study indicates that children with asthma have impaired innate immune responses, which may explain the high frequency of viral-induced acute exacerbations in this population. Furthermore, asthma was associated with increased frequency of ILC subsets, which could contribute to airway inflammation and tissue remodelling. Increased understanding of innate immune responses may facilitate the development of therapeutic strategies to prevent acute asthma exacerbations in young children with asthma.

## Data Availability Statement

The datasets presented in this study can be found in online repositories. The names of the repository/repositories and accession number(s) can be found below: https://ogma.newcastle.edu.au/vital/access/%20/manager/Repository/uon:37141.

## Ethics Statement

The studies involving human participants were reviewed and approved by HNEH Ethics Committee (15/06/17/4.03). Written informed consent to participate in this study was provided by the participants’ legal guardian/next of kin.

## Author Contribution

LW, BB, MS, AC, MJ, and PW were involved in design of the study. BB, BH, and RM conducted the study. KN was involved in developing the laboratory methods for PBMC isolating and culture. MS, AC, and BH were involved in developing laboratory methods for cell quantification. BH and KN performed the experiments. BH performed the analysis and drafted the manuscript with input from BB, MS, AC, and LW. BB and LW verified the analytical methods. All authors discussed the results and commented on the manuscript. All authors contributed to the article and approved the submitted version.

## Funding

This study has been funded by Hunter Medical Research Institute Gastronomic Lunch project grant (G1500957). Funding sources had no role in the study design, collection, analysis, and interpretation of data or in the decision to submit or writing the manuscript.

## Conflict of Interest

The authors declare that the research was conducted in the absence of any commercial or financial relationships that could be construed as a potential conflict of interest.
