# Supplementary material for: Children With Asthma Have Impaired Innate Immunity and Increased Numbers of Type 2 Innate Lymphoid Cells Compared With Healthy Controls
Source: Front Immunol. 2021 Jun 17;12:664668. doi: 10.3389/fimmu.2021.664668 (PMC8248177; doi:10.3389/fimmu.2021.664668)
Supplement: Supplementary file 1 [file DataSheet_1.docx]

**Supplementary Materials**

**METHODS**

Inclusion criteria for asthmatics

Recent exacerbation/s (≥1 in 6 or ≥2 in past 12 months) that resulted in OCS use or an unscheduled asthma healthcare visit (GP, emergency department (ED) or admission). Stable asthma was defined as no change in asthma medications, unscheduled asthma healthcare visit, use of OCS or antibiotics in past 4 weeks.

Medical history

Medical, family, exacerbation and medication history were collected.

Anthropometry

Body weight was measured in light clothing without shoes, using a digital scale to the nearest 100g (NU WEIGH LOG842 scales, NU Weigh Scales Inc, MI). Height was measured to the nearest 0.1cm without shoes, using the stretch stature method and a wall-mounted stadiometer. BMI z-scores were calculated with reference to the Centre for Disease Control and Prevention (CDC) 2000 Growth Charts, using an online calculator.

Impulse Oscillometry (IOS) Measurements

IOS measurements were performed as per the ATS/ERS recommendations^1^ using the Jaeger MasterScreen IOS system (Jaeger Co, Hoechberg, Germany), which was calibrated daily using a 3litre syringe as per manufacturer’s specifications, by trained operators. Each participant wore a nose clip and was seated whilst performing the manoeuvre. A new disposable filter with mouthpiece was attached to the pneumotach, and the participant was instructed to inhale and exhale normally through the mouthpiece until completion of the test (30 seconds). This was repeated until three reproducible and technically correct results were obtained, with a maximum of eight attempts performed. Results are reported as the mean value of at least 2 reproducible tests (coefficient of variation of at least two tests <10%) with coherence values >0.8 at 5Hz and between 0.9-1.0 at 20Hz. The operator evaluated each test to ensure efforts were free from artefacts caused by coughing, swallowing, vocalisation or breath holding. Mean values of resistance (R) and reactance (X) at 5, 10 and 20 Hz, the area of reactance (Ax) were calculated and reference values from Dencker and Malmberg et al (2006) were used to generate z-scores for analysis^2^.

Nitrogen Multiple-Breath Washout (N_2_MBW)

N_2_MBW was assessed with at least two trials (ideally three) using an open circuit bias flow system (Exhalyzer D, EcoMedics AG, Switzerland), and Spiroware 3.1.6 software (EcoMedics AG, Switzerland). Nitrogen (N2) concentration was measured indirectly by measuring oxygen (O2) and carbon dioxide (CO2) concentrations (Capnostat 5, Philips Healthcare, the Netherlands) simultaneously. Flow and gas calibration were performed daily as per manufacturers’ instructions. Children were asked to perform normal breathing whilst sitting in an upright position using a facemask fitted with Thera-putty to prevent leaks while watching an appropriate movie. Once an acceptable steady tidal breath pattern was established with a stable end expiratory lung volume, the washout phase was commenced during which the subject breathed 100% O_2_ until end-tidal N_2_ concentrations reached below 1/40th of the original starting concentration^3^. Trials were retrospectively evaluated for stable tidal volume (at least 5 stable breaths) before the start of washout phase, evidence of sighs and very small breaths at the beginning of washout phase, and evidence of a leak. If evidence of these factors were present trials were deemed technically unacceptable^4^. Lung Clearance Index (LCI) was calculated as the cumulative expired volume (CEV) divided by functional residual capacity (FRC) (LCI = CEV/FRC)^3^. LCI was determined as the average of at least two technically acceptable tests, defined as a regular tidal pattern throughout the test, no signs of leaks and values lying within 10% of each other^5^. Repeatability of FRC was acceptable if the mean between technically accepted trials were within 25%^4^.

PBMC isolation and culture

PBMCs were resuspended in Roswell Park Memorial Institute media (Invitrogen, Australia Pty Limited), 5% foetal bovine serum (Invitrogen, Australia Pty Limited). Cell viability was evaluated by trypan blue staining, which differentiates live cells from dead cells based on cellular necrosis. Cell counts were performed. PBMCs were then adjusted to a concentration of 1.0 x 10^6^ viable cells per well in 24-well plates (sterile, tissue culture grade, Australia) and cultured with medium alone (control) or with RV-1β at a final concentration of 20 multiplicity of infection, or HDM (Greer Laboratories, USA) at final concentration of 100ug/mL^6^ or LPS (Sigma-Aldrich, USA) at a final concentration of 100ug/mL^7^ for a total of 48h, at 33^o^C and 5% CO_2_^8^. The media was not changed during this period. Cellular suspensions were centrifuged at 550Xg for 10 min and supernatants stored at -80^o^C for subsequent analyses. Experimental protocol and procedures were approved by the University of Newcastle Biosafety Committee (approval number R5/2017).

Cytokine assays

The concentrations of IFN-γ, IL-1β and IL-6 in the culture supernatants were analysed using bead-based multiplex assay (BD Bioscience, Sydney, Australia). The assay range was 10.0-2500 pg/ml. The minimum detection limit of this assay was 1.8 pg/ml. IFN-λ and IL-5 concentrations in the culture supernatants were measured using a high-sensitivity commercial ELISA assay (R&D Systems, Sydney, Australia), as per the manufacturer’s recommendations. The assay range was 50-4000 pg/ml. The minimal detectable dose was ≤10 pg/ml. All samples were tested in duplicates and the %CV between duplicate samples was accepted when <10%.

Whole blood flow cytometry

Immune-phenotyping was performed in whole blood using a lyse-wash procedure, where red blood cells are removed after staining with antibodies by targeted red blood cell lysis, leaving the leukocytes for analysis. A list of antibodies used is presented in **Table S1**. Cell subsets were predetermined based on the specific surface markers previously identified in the literature ^9,10^ **(Table S2)**. Cell events were gated by using a predefined gating strategy (Figure S1). ILC and granulocytes were adjusted for x10^6^ CD45^+^ cells. DCs were adjusted for x10^6^ human leukocyte antigen^+^ cells. Lymphocytes and NK cells were adjusted for x10^6^ CD3^+^ cells.

| **Table S1- Antibodies for flow cytometric analysis of whole blood immune phenotypes** | | | | |
| --- | --- | --- | --- | --- |
| **Antigen** | **Fluorochrome** | **Clone** | **Product code** | **Source** |
| **Innate lymphoid cells** | | | | |
| TCR-αβ | PE | H57-597 | 563221 | BD Biosciences |
| TCR-γδ | PE | GL3 | 563218 | BD Biosciences |
| CD19 | PE | HIB19 | 561741 | BD Biosciences |
| CD11c | PE | HL3 | 561044 | BD Biosciences |
| CD94 | PE | HP-3D9 | 555889 | BD Biosciences |
| CD14 | PE | rmC5-3 | 553740 | BD Biosciences |
| CD1a | PE | HI149 | 555807 | BD Biosciences |
| CD34 | PE | RAM34 | 551387 | BD Biosciences |
| CD123 | PE | 7G3 | 561058 | BD Biosciences |
| FceRa1 | PE | AER-37 | 334610 | Biolegend |
| CD303 | PE | 201A | 354204 | Biolegend |
| CD117 (c-kit) | PE-Cy7 | 104D2 | 339195 | BD Biosciences |
| CD45 | APC-Cy7 | 2D1 | 348795 | BD Biosciences |
| CD3 | BV510 | UCHT1 | 563109 | BD Biosciences |
| CD161 | BV711 | HP-3G10 | 748282 | BD Biosciences |
| CD336 (NKp44) | BB515 | p44-8 | 565099 | BD Biosciences |
| CD127 | BV421 | HIL-7R-M21 | 562436 | BD Biosciences |
| CD294 (CRTH2) | BV786 | BM16 | 741016 | BD Biosciences |
| **Neutrophils and eosinophils** | | | | |
| CD16 | BV421 | 3G8 | 562874 | BD Biosciences |
| CD193 | PE | 5E8 | 561746 | BD Biosciences |
| CD45 | APC | H130 | 555485 | BD Biosciences |
| **T lymphocytes** | | | | |
| CD127 | BV421 | HIL-7R-M21 | 562436 | BD Biosciences |
| TCR αβ | BV510 | T10B9 | 563625 | BD Biosciences |
| TCR γδ | FITC | B1 | 561995 | BD Biosciences |
| CD25 | PE | M-A251 | 557138 | BD Biosciences |
| CD3 | PE-Cy7 | UCHT1 | 555334 | BD Biosciences |
| CD4 | APC | RPA-T4 | 561840 | BD Biosciences |
| CD8 | APC-CY7 | Sk1 | 560273 | BD Biosciences |
| **Dendritic cells** | | | | |
| CD16 | BV421 | 3G8 | 562874 | BD Biosciences |
| HLA-DR | BV510 | G46-6 | 563083 | BD Biosciences |
| CD1c | BB515 | F10/21A3 | 565054 | BD Biosciences |
| CD141 | PE | 1A4 | 559781 | BD Biosciences |
| CD14 | PerCP-Cy5.5 | M5E2 | 561116 | BD Biosciences |
| CD303 | APC | 201A | 354206 | Biolegend |
| CD19 | PE-Cy7 | HIB19 | 560728 | BD Biosciences |
| CD3 | PE-Cy7 | UCHT1 | 555334 | BD Biosciences |
| CD56 | PE-Cy7 | B159 | 557747 | BD Biosciences |
| **Natural killer cells and B cells** | | | | |
| CD56 | PE-Cy7 | B159 | 557747 | BD Biosciences |
| CD3 | BV510 | UCHT1 | 563109 | BD Biosciences |
| CD19 | PE | HIB19 | 561741 | BD Biosciences |
| CD16 | BV421 | 3G8 | 562874 | BD Biosciences |
| CD161 | BV711 | HP-3G10 | 748282 | BD Biosciences |
| CD8 | APC-CY7 | Sk1 | 560273 | BD Biosciences |
| TCR Vα24-Jα18 | FITC | 6B11 | 558371 | BD Biosciences |
| CD14 | Per-CP Cy5.5 | M5E2 | 561116 | BD Biosciences |

Abbreviations: APC, Allophycocyanin; BB, brilliant blue; BV, brilliant violet; FITC, fluorescein isothiocyanate; HLA, human leukocyte antigen; PE, phycoerythrin; PerCP, peridinin-chlorophyll-protein complex; TCR, T cell receptor

| **Table S2- Surface markers used to identify each cell subset** | |
| --- | --- |
| **Cell type** | **Surface markers** |
| Leukocytes | CD45^+^ |
| Granulocytes | CD45^+^ SSC ^int/high^ |
| Eosinophils | CD45^+^ SSC ^int/high^ CD193^high^ |
| Neutrophils | CD45^+^ SSC ^int/high^ CD193^low^ |
| T cells | CD3^+^ |
| CD4 T cells | αβ TCR^+^ CD4^+^ |
| CD8 T cells | αβ TCR^+^ CD8^+^ |
| Activated CD4 T cells | αβ TCR^+^ CD4^+^ CD25^+^ CD127 ^high/+^ |
| Activated CD8 T cells | αβ TCR^+^ CD8^+^ CD25^+^ CD127 ^high/+^ |
| Treg cells | αβ TCR^+^ CD4^+^ CD25^+^ CD127 ^low/-^ |
| γδ T cells | γδ TCR^+^ CD4^+^ |
| B cells | CD3^-^ CD19^+^ |
| NK cells | CD3^-^ CD56^+^ CD16^+^ |
| BDCA-1 DCs | CD3^-^ CD19^-^ CD56^-^ CD14^-^ CD16^-^ BDCA-1^+^ |
| BDCA-3 DCs | CD3^-^ CD19^-^ CD56^-^ CD14^-^ CD16^+^ CD1c^-^ CD303^-^ BDCA-3^high^ |
| pDCs | CD3^-^ CD19^-^ CD56^-^ CD14^-^ BDCA-2^+^ |
| ILC1s | CD45^+^ Human lineage^1-^ CD161^+^ CRTH2^-^ CD117^-^ NKp44^-^ |
| ILC2 | CD45^+^ Human lineage^-^ CD161^+^ CRTH2^+^ |
| NCR^-^ILC3s | CD45^+^ Human lineage^-^ CD161^+^ CRTH2^-^ CD117^+^ NKp44^-^ |
| NCR^+^ ILC3s | CD45^+^ Human lineage^-^ CD161^+^ CRTH2^-^ CD117^+^ NKp44^+^ |
| ^1^The lineage cocktail covered markers for T cells (TCRαβ and TCR γδ), B cells (CD19), NK cells (CD94), myeloid and plasmacytoid dendritic cells (CD1a, CD11c, CD123, and BDCA-2), monocytes and macrophages (CD14), mast cells (FceR1), and stem cells (CD34). Abbreviations: BDCA, blood dendritic cell antigen; DCs, dendritic cells; ILCs, innate lymphoid cells; NCR, natural cytotoxic receptor; NK, natural killer; TCR**,** T-cell receptor; Treg, regulatory T cells; pDCs, plasmacytoid dendritic cells | |

**RESULTS**

| **Table S3- Cell supernatant cytokine secretion of PBMCs in response to different stimuli in children with asthma and age-matched non-asthmatic group with negative RAST results** | | | | |
| --- | --- | --- | --- | --- |
| **Cytokine** *(pg/ml)* | **Asthma (n=19)** | | **Healthy controls (n=13)** | ***P*-value** |
| *PBMCs stimulated with Rhinovirus-1* | | | | |
| **IFN-γ** | 14.79 (6.33, 46.11) | 41.23 (22.56, 110.71) | | **0.018** |
| **IFN-λ** | 10.68 (2.53, 100.07) | 6.10 (3.18, 91.07) | | 0.921 |
| **IL-1β** | 29.76 (5.53, 109.33) | 7.92 (1.75, 23.01) | | 0.057 |
| **IL-5** | 0.99 (0.44, 1.64) | 0.91 (0.38, 1.56) | | 0.702 |
| **IL-6** (*ng/mL)* | 1.81 (0.30, 9.90) | 0.45 (0.18, 3.42) | | 0.495 |
| *PBMCs stimulated with House Dust Mite* | | | | |
| **IFN-γ** | 1.14 (0.77, 1.43) | 0.98 (0.77, 1.26) | | 0.466 |
| **IFN-λ** | 2.43 (1.00, 5.11) | 3.42 (1.76, 5.76) | | 0.466 |
| **IL-1β** | 13.78 (9.81, 37.26) | 63.84 (45.67, 147.20) | | **0.002** |
| **IL-5** | 1.20 (0.64, 12.24) | 0.88 (0.51, 1.40) | | 0.103 |
| **IL-6** (*ng/mL)* | 6.30 (0.94, 17.19) | 31.47 (16.89, 60.097) | | **0.001** |
| *PBMCs stimulated with Lipopolysaccharide* | | | | |
| **IFN-γ** | 2.18 (1.14, 16.05) | 31.03 (15.54, 55.44) | | **0.009** |
| **IFN-λ** | 3.35 (1.25, 7.43) | 2.12 (1.27, 2.73) | | 0.219 |
| **IL-1β** | 2042.31 (1249.20, 8509.17) | 10864.20 (5722.54, 17698.95) | | **0.005** |
| **IL-5** | 0.57 (0.22, 0.82) | 0.62 (0.30, 0.90) | | 0.699 |
| **IL-6** (*ng/mL)* | 96.70 ± 53.32 | 158.82 ± 136.82 | | 0.082 |
| Data are presented as median (interquartile) or mean ± SD. All variables adjusted for the levels in uninfected PBMCs (control). Difference between groups analysed by Wilcoxon Rank Sum test (non-parametric data) or two-sample t-test (parametric data). PBMC, peripheral blood mononuclear cells; IFN, interferon; IL, interleukin. | | | | |

| **Table S4- Cell supernatant cytokine secretion of PBMCs in response to different stimuli in children with asthma and age-matched non-asthmatic group with no history of OCS use** | | | |
| --- | --- | --- | --- |
| **Cytokine** *(pg/ml)* | **Asthma (n=27)** | **Healthy controls (n=14)** | ***P*-value** |
| *PBMCs stimulated with Rhinovirus-1* | | | |
| **IFN-γ** | 14.09 (6.33, 28.63) | 39.40 (26.57, 105.40) | **0.003** |
| **IFN-λ** | 22.69 ± 50.66 | 45.71 ± 72.97 | 0.244 |
| **IL-1β** | 65.30 ± 139.67 | 18.02 ± 25.62 | 0.255 |
| **IL-5** | 0.89 (0.36, 1.57) | 0.85 (0.31, 1.48) | 0.713 |
| **IL-6** (*ng/mL)* | 3.64 ± 7.27 | 9.79 ± 30.20 | 0.317 |
| *PBMCs stimulated with House Dust Mite* | | | |
| **IFN-γ** | 1.01 ± 0.49 | 1.18 ± 0.78 | 0.404 |
| **IFN-λ** | 2.98 ± 2.51 | 3.97 ± 2.13 | 0.217 |
| **IL-1β** | 13.78 (4.89, 44.77) | 76.99 (49.27, 133.17) | **0.001** |
| **IL-5** | 22.74 ± 35.65 | 13.33 ± 46.06 | 0.473 |
| **IL-6** (*ng/mL)* | 6.30 (1.07, 12.74) | 30.90 (18.24, 58.45) | **<0.0001** |
| *PBMCs stimulated with Lipopolysaccharide* | | | |
| **IFN-γ** | 1.50 (1.08, 5.16) | 41.14 (15.79, 64.18) | **<0.0001** |
| **IFN-λ** | 5.10 ± 7.74 | 2.27 ± 1.08 | 0.184 |
| **IL-1β** | 1780 (930, 3990) | 10680 (6470, 16850) | **<0.0001** |
| **IL-5** | 0.61 (0.24, 0.85) | 0.67 (0.35, 0.94) | 0.887 |
| **IL-6** (*ng/mL)* | 86.86 ± 50.17 | 152.96 ± 133.27 | **0.029** |
| Data are presented as median (interquartile) or mean ± SD. All variables adjusted for the levels in uninfected PBMCs (control). Difference between groups analysed by Wilcoxon Rank Sum test (non-parametric data) or two-sample t-test (parametric data). OCS, oral corticosteroids; PBMC, peripheral blood mononuclear cells; IFN, interferon; IL, interleukin. | | | |

**Figure Legends**

Figure S1- Gating strategy used to identify each immune cell subset


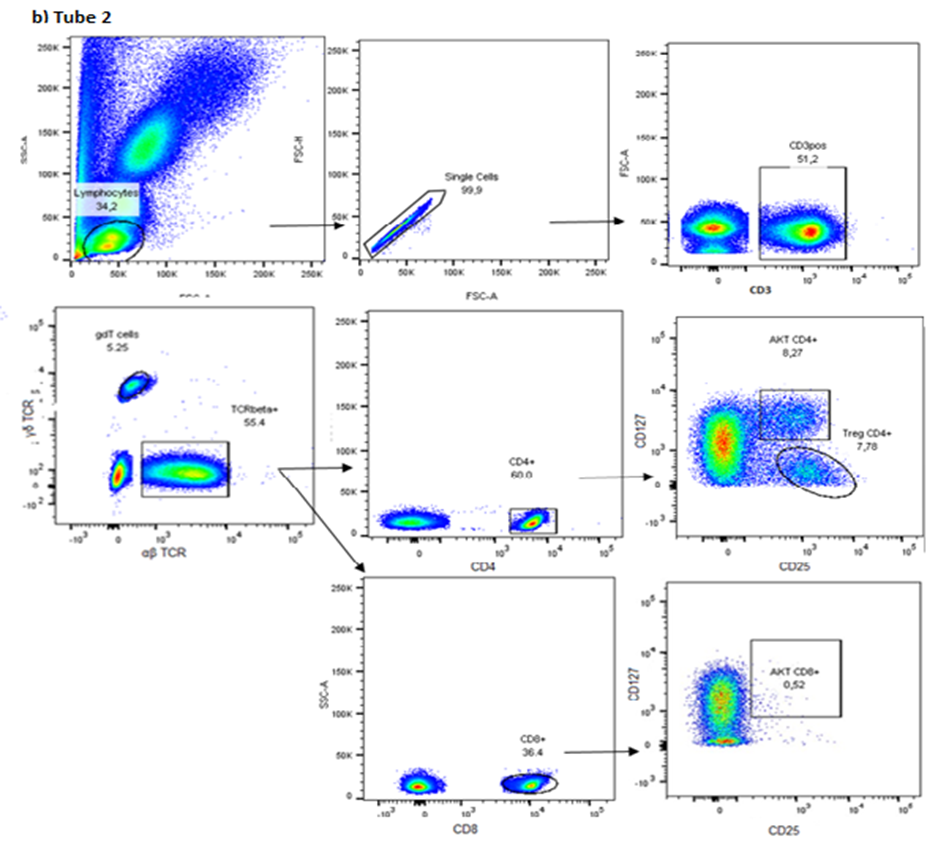

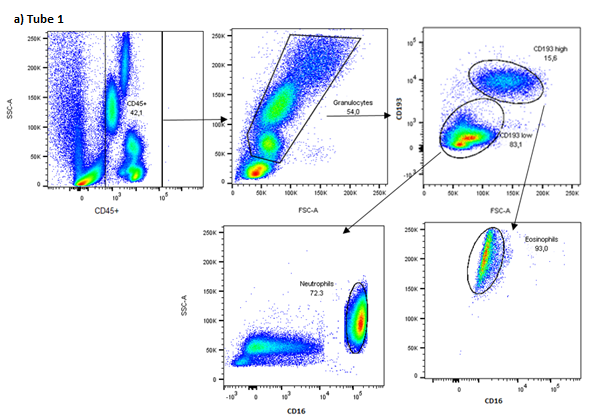


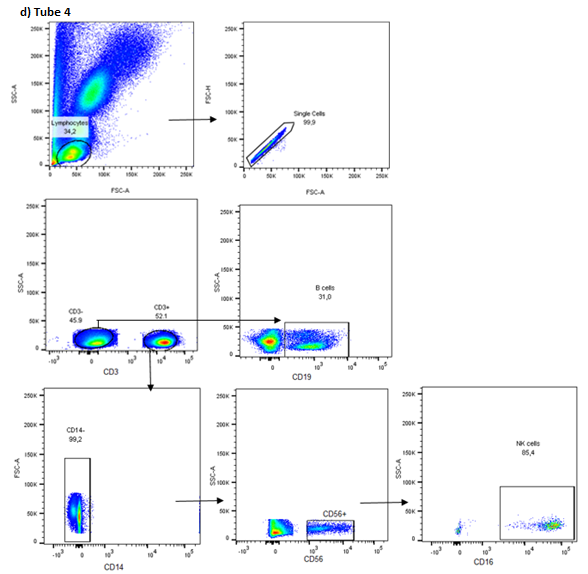

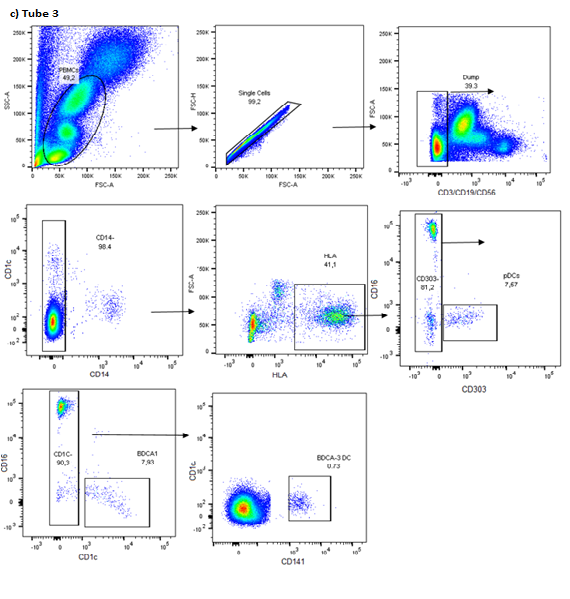


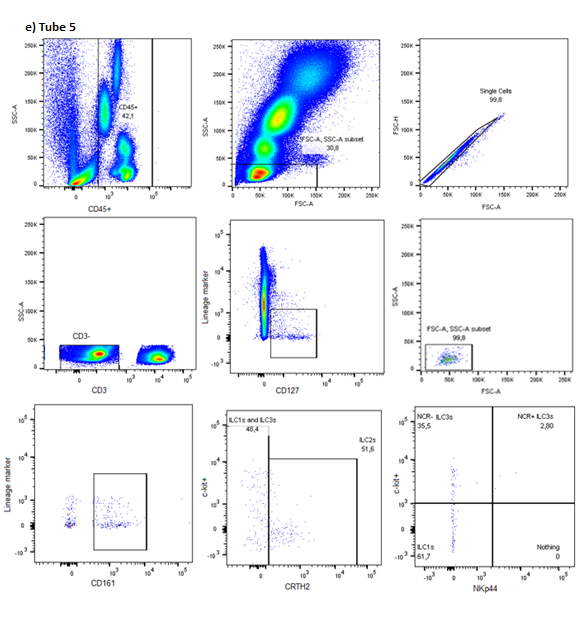


Figure S1

**References**

1. Beydon N, Davis SD, Lombardi E, et al. An official American Thoracic Society/European Respiratory Society statement: Pulmonary function testing in preschool children. American Journal of Respiratory and Critical Care Medicine 2007;175(12):1304-1345. DOI: 10.1164/rccm.200605-642ST.

2. Dencker M, Malmberg LP, Valind S, et al. Reference values for respiratory system impedance by using impulse oscillometry in children aged 2-11 years. Clinical physiology and functional imaging 2006;26(4):247-50. (In eng). DOI: 10.1111/j.1475-097X.2006.00682.x.

3. Robinson PD, Latzin P, Verbanck S, et al. Consensus statement for inert gas washout measurement using multiple- and single- breath tests. European Respiratory Journal 2013;41(3):507-522. (In eng). DOI: 09031936.00069712

4. Jensen R, Stanojevic S, Klingel M, et al. A systematic approach to multiple breath nitrogen washout test quality. PLOS ONE 2016;11(6):e0157523. (<https://doi.org/10.1371/journal.pone.0157523>).

5. Robinson PD, Stocks J, Aurora P, Lum S. Abbreviated multi-breath washout for calculation of lung clearance index. Pediatric Pulmonology 2013;48(4):336-343. (In eng). DOI: 10.1002/ppul.22618 [doi].

6. Mishra A, Brown AL, Yao X, et al. Dendritic cells induce Th2-mediated airway inflammatory responses to house dust mite via DNA-dependent protein kinase. Nature communications 2015;6:6224. (In eng). DOI: 10.1038/ncomms7224.

7. Plevin RE, Knoll M, McKay M, Arbabi S, Cuschieri J. The Role of Lipopolysaccharide Structure in Monocyte Activation and Cytokine Secretion. Shock 2016;45(1):22-7. (In eng). DOI: 10.1097/shk.0000000000000470.

8. Forbes RL, Gibson PG, Murphy VE, Wark PA. Impaired type I and III interferon response to rhinovirus infection during pregnancy and asthma. Thorax 2012;67(3):209-14. (In eng). DOI: 10.1136/thoraxjnl-2011-200708.

9. Hazenberg MD, Spits H. Human innate lymphoid cells. Blood 2014;124(5):700-9. (In eng). DOI: 10.1182/blood-2013-11-427781.

10. Thysen AH, Rasmussen MA, Kreiner-Moller E, et al. Season of birth shapes neonatal immune function. J Allergy Clin Immunol 2016;137(4):1238-46.e1-13. (In eng). DOI: 10.1016/j.jaci.2015.08.041.
